# Dual targeting of the androgen receptor and PI3K/AKT/mTOR pathways in prostate cancer models improves antitumor efficacy and promotes cell apoptosis

**DOI:** 10.1002/1878-0261.13577

**Published:** 2024-01-15

**Authors:** Tatsuo Sugawara, Ekaterina Nevedomskaya, Simon Heller, Annika Böhme, Ralf Lesche, Oliver von Ahsen, Sylvia Grünewald, Holly M. Nguyen, Eva Corey, Simon J. Baumgart, Victoria Georgi, Vera Pütter, Amaury Fernández‐Montalván, James D. Vasta, Matthew B. Robers, Oliver Politz, Dominik Mumberg, Bernard Haendler

**Affiliations:** ^1^ Bayer AG, Pharmaceuticals, Research & Early Development Oncology Berlin Germany; ^2^ Nuvisan ICB GmbH Berlin Germany; ^3^ Department of Urology University of Washington Seattle WA USA; ^4^ Promega Corporation Madison WI USA; ^5^ Present address: Boehringer Ingelheim Pharma GmbH & Co. KG Biberach an der Riß Germany; ^6^ Present address: Adcento ApS Copenhagen Denmark

**Keywords:** androgen receptor, PI3 kinase, Prostate

## Abstract

Prostate cancer is a frequent malignancy in older men and has a very high 5‐year survival rate if diagnosed early. The prognosis is much less promising if the tumor has already spread outside the prostate gland. Targeted treatments mainly aim at blocking androgen receptor (AR) signaling and initially show good efficacy. However, tumor progression due to AR‐dependent and AR‐independent mechanisms is often observed after some time, and novel treatment strategies are urgently needed. Dysregulation of the PI3K/AKT/mTOR pathway in advanced prostate cancer and its implication in treatment resistance has been reported. We compared the impact of PI3K/AKT/mTOR pathway inhibitors with different selectivity profiles on *in vitro* cell proliferation and on caspase 3/7 activation as a marker for apoptosis induction, and observed the strongest effects in the androgen‐sensitive prostate cancer cell lines VCaP and LNCaP. Combination treatment with the AR inhibitor darolutamide led to enhanced apoptosis in these cell lines, the effects being most pronounced upon cotreatment with the pan‐PI3K inhibitor copanlisib. A subsequent transcriptomic analysis performed in VCaP cells revealed that combining darolutamide with copanlisib impacted gene expression much more than individual treatment. A comprehensive reversal of the androgen response and the mTORC1 transcriptional programs as well as a marked induction of DNA damage was observed. Next, an *in vivo* efficacy study was performed using the androgen‐sensitive patient‐derived prostate cancer (PDX) model LuCaP 35 and a superior efficacy was observed after the combined treatment with copanlisib and darolutamide. Importantly, immunohistochemistry analysis of these treated tumors showed increased apoptosis, as revealed by elevated levels of cleaved caspase 3 and Bcl‐2‐binding component 3 (BBC3). In conclusion, these data demonstrate that concurrent blockade of the PI3K/AKT/mTOR and AR pathways has superior antitumor efficacy and induces apoptosis in androgen‐sensitive prostate cancer cell lines and PDX models.

## Graphical abstract

Crosstalk between the androgen receptor (AR) and the PI3K/AKT/mTOR pathways plays an essential role in prostate cancer growth and therapy resistance. Treatment with the AR inhibitor darolutamide and the pan‐PI3K inhibitor copanlisib strongly reduces survival and growth of prostate cancer models.

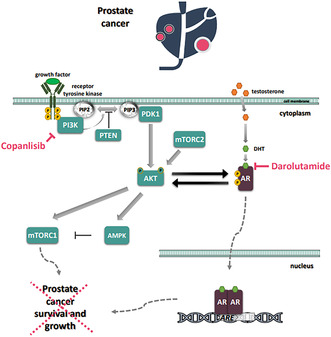

**Abbreviations**
ACSL3: acyl CoA synthetase long‐chain family member 3
AR: androgen receptor
BBC3: Bcl‐2‐binding component 3
CI: combination index
CRPC: castration‐resistant prostate cancer
DHCR7: 7‐dehydrocholesterol reductase
DMSO: dimethyl sulfoxide
EBP: emopamil‐binding protein
EIF: eukaryotic translation initiation factor
ELOVL6: elongation of very long chain fatty acid protein 6
FDR: false discovery rate
FKBP5: FK506‐binding protein prolyl isomerase 5
IHC: immunohistochemistry
kPCA: kinetic probe competition assay
mCRPC: metastatic CRPC
mTOR: mammalian target of rapamycin
PARP: poly(ADP‐ribose) polymerase
PCA: principal component analysis
PDX: patient‐derived xenograft
PI3K: phosphatidylinositol 3‐kinase
PSMA: prostate‐specific membrane antigen
PTEN: phosphatase and tensin homolog
SPR: surface plasmon resonance
ssGSEA: single‐sample gene set enrichment analysis
TMPRSS2: transmembrane protease, serine 2
TPM: transcripts per million

## 1 Introduction

Prostate cancer is among the most frequent cancer types in men worldwide with a probability of one in eight men of being affected and a total of 288 300 new cases being expected in the United States in 2023 [1]. Local irradiation, prostatectomy, and androgen deprivation therapy are highly effective treatments of confined disease [2]. However, castration‐resistant prostate cancer (CRPC) often emerges after a few years, as reflected by elevated serum levels of prostate‐specific antigen (PSA). This disease stage is usually treated with androgen receptor (AR) signaling inhibitors, which act by competitive antagonism or by steroid synthesis blockade [3]. This therapy will significantly delay tumor progression and prolong overall survival, but metastatic CRPC (mCRPC) will ultimately develop [4]. Resistance mechanisms include AR overexpression, appearance of AR mutations and splice variants, as well as increased androgen synthesis in prostate cancer cells, indicating that aberrant AR signaling still remains an essential player in late‐stage tumors [5, 6].

Drugs addressing other signaling pathways are now also marketed for mCRPC. The taxanes docetaxel and cabazitaxel are approved for late‐stage prostate cancer, and the combination of docetaxel with androgen deprivation therapy and the AR inhibitor darolutamide has recently been shown to have superior clinical benefits [7, 8]. Two poly(ADP‐ribose) polymerase (PARP) inhibitors are used to treat men with mCRPC harboring alterations in homologous recombination repair genes [9]. Combination studies of PARP inhibitors with different AR signaling inhibitors are currently being clinically assessed [9]. The α‐emitter radium‐223 is approved for mCRPC patients with bone metastases, but no visceral involvement [10], and the cotreatment with the AR inhibitor enzalutamide is being evaluated in the clinic [11]. The prostate‐specific membrane antigen (PSMA)‐binding drug ^177^Lu‐PSMA‐617 is approved for second‐line treatment of mCRPC patients positive for PSMA, and additional PSMA‐targeting agents coupled to α‐ or β‐emitters are being evaluated in the clinic [12].

Several studies document that the phosphatidylinositol 3‐kinase (PI3K)/AKT/mammalian target of rapamycin (mTOR) pathway plays an essential role in prostate cancer. Loss of the phosphatase and tensin homolog (PTEN) tumor suppressor gene causing uncontrolled activation of the PI3K/AKT/mTOR pathway is observed in numerous patients [13]. Also, amplification and activating mutations of PI3K, mainly the alpha isoform, have been reported in late‐stage prostate cancer [13, 14, 15]. Small molecules blocking the PI3K/AKT/mTOR pathway include pan‐ and selective PI3K or AKT isoform inhibitors, dual PI3K/mTOR inhibitors, and mTOR inhibitors [16, 17, 18]. More recently, compounds addressing mutant forms of PI3Kα have been described [17, 18]. PI3K/AKT/mTOR inhibitors have been clinically evaluated and in some cases approved, mainly for leukemia and lymphoma [18, 19]. However, clinical studies performed in prostate cancer using PI3K/AKT/mTOR inhibitors with different selectivity profiles have shown only limited efficacy, mainly due to relief of feedback inhibition [20] and adverse events observed at effective doses [21, 22].

The reciprocal feedback between the AR and the PI3K/AKT/mTOR pathways leading to prostate tumor survival and progression has been reported [23, 24]. Inhibition of PI3K signaling activates AR function, whereas AR blockade stimulates AKT [23]. Combining an AR inhibitor with a compound targeting the PI3K/AKT/mTOR pathway may therefore increase the efficacy of anti‐androgen treatment, and first combination approaches are currently being assessed in the clinic [18, 21, 25]. Early data suggest AKT inhibition to be superior to PI3K inhibition in the context of PTEN loss [26]. However, a phase 3 trial combining the AKT inhibitor ipatasertib with the androgen biosynthesis inhibitor abiraterone failed to show a significant overall survival improvement in an interim analysis, and numerous adverse events were reported [27]. The AKT inhibitor capivasertib combined with abiraterone is currently in a phase 3 trial in metastasized hormone‐sensitive prostate cancer patients with PTEN loss [28]. Another phase 3 investigation has just been started combining capivasertib with docetaxel, based on a phase 2 study showing this approach to improve overall survival in mCRPC patients [29]. Concerning the efficacy of PI3K inhibitors in prostate cancer, studies are still at an early stage. A phase 2 trial combining the pan‐PI3K inhibitor buparlisib with enzalutamide for therapy of mCRPC failed to show benefit [20]. More recently, a phase 2 study evaluating the PI3K, mTOR, and DNA protein kinase inhibitor samotolisib together with enzalutamide revealed improvement in progression‐free survival, mainly in mCRPC patients with intact PTEN and no AR‐V7 splice variant [30]. The pan‐PI3K inhibitor copanlisib is currently being evaluated in mCRPC patients in a phase 1b/2 study in combination with the PARP inhibitor rucaparib, and a clinical benefit rate was achieved [31]. Studies with isoform‐selective PI3K inhibitors were generally less encouraging. The PI3Kβ/δ‐selective inhibitor AZD8186 underwent a phase 1 study as single agent and in combination with abiraterone or with the mTOR inhibitor vistusertib. It showed limited activity in patients with PTEN‐deficient or mutated mCRPC [21]. Minor impact was also reported for the combination of the PI3Kβ‐selective inhibitor GSK2636717 with enzalutamide [32]. Concerning mTOR inhibitors, phase 2 trials for everolimus, temsirolimus, and sapanisertib in CRPC have been completed, but limited efficacy was observed due to dose limitations linked to toxicity [33, 34, 35]. An earlier phase 2 study combining everolimus with bicalutamide also reported substantial side effects [36].

In view of the adverse events reported for PI3K/AKT/mTOR pathway inhibitors in clinical studies, it will be essential to determine which selectivity profile is best suited for prostate cancer therapy and has the optimal potential for combination with drugs targeting AR signaling. In this study, we first compared the target‐binding affinities and kinetic parameters of different PI3K inhibitors and their antiproliferative impact on five different prostate cancer cell lines. We then investigated the pro‐apoptotic effect of these compounds on the most responsive cell lines, as single agents or in combination with the recently approved AR inhibitor darolutamide [7], a nonsteroidal compound with a unique chemical structure [37, 38] and favorable safety profile [39]. We found the pan‐PI3K inhibitor copanlisib [40] to be one of the most effective compounds and its combination potential with darolutamide was then studied in detail in the VCaP prostate cancer cell line. Reversal of the androgen‐response and of the mTORC1 transcriptional programs as well as induction of DNA damage response were observed. An *in vivo* efficacy study conducted in the patient‐derived xenograft (PDX) model LuCaP 35 [41] furthermore showed superior efficacy of the combination treatment and stimulation of apoptosis.

## 2 Materials and methods

### 2.1 Compounds

R1881 (synthetic androgen, also known as metribolone) and copanlisib were manufactured in‐house. Darolutamide was synthesized at Orion Corporation (Espoo, Finland). Apitolisib (S2696), dactolisib (S1009), capivasertib (S8019), and camptothecin (S1288) were from Selleckchem (Planegg, Germany). Alpelisib (T1921), duvelisib (T1988), and idelalisib (T1894) were from TargetMol (Wellesley Hills, MA, USA). Ipatasertib (HY‐15186) was from MedChemExpress (Monmouth Junction, NJ, USA).

### 2.2 Cell lines

The VCaP (RRID:CVCL_2235), LNCaP (RRID:CVCL_0395), 22Rv1 (RRID:CVCL_1045), PC‐3 (RRID:CVCL_0035), DU145 (RRID:CVCL_0105), and HeLa (RRID:CVCL_0030) cancer cell lines were acquired from the ATCC (American Type Culture Collection, Manassas, VA, USA). DNA fingerprinting was performed at the DSMZ (Deutsche Sammlung von Mikroorganismen und Zellkulturen, Braunschweig, Germany) for authentication. Mycoplasma contamination testing was carried out using MycoAlert (Lonza, Cologne, Germany).

### 2.3 Determination of target binding affinity and kinetic parameters

The principle of the homogeneous kinetic probe competition assay (kPCA) has been described [42], and detailed protocols for PI3Kα reported [43]. For the surface plasmon resonance (SPR) assay, PI3Kα was captured on a Biacore streptavidin‐CM5 chip and compound binding at increasing concentrations was analyzed in real time with a Biacore T200 SPR instrument (both from GE Healthcare Life Sciences, Uppsala, Sweden). Compound association and dissociation traces were fitted to a 1 : 1 Langmuir model to obtain the *k*_on_ and *k*_off_ kinetic parameters of the interaction. Steady‐state resonance unit signals from the same experiment were plotted against the compound dose to calculate the equilibrium affinity using a 1 : 1 binding model derived from the law of mass action.

The intracellular inhibitor potency for PI3Kα was evaluated using a probe displacement assay based on the NanoBRET technology [44]. HeLa cells transiently expressing NanoLuc‐labeled PI3Kα and cotransfected with the p85 regulatory subunit were equilibrated with increasing compound concentrations in the presence of a fixed amount (40 nm) of the Kinase Tracer 03 (Promega Corporation, Madison, WI, USA). Following the addition of the compounds and tracer, the cells were incubated for 3 h before the addition of Complete NanoBRET Nano‐Glo substrate plus extracellular NanoLuc inhibitor (both from Promega Corporation). Subsequent bioluminescence resonance energy transfer measurements were made on a GloMax Discover instrument (Promega Corporation). Competitive displacement curves for the test compound were fitted to the sigmoidal dose–response function to calculate apparent intracellular IC_50_ values at each tracer concentration. The apparent intracellular IC_50_ values were replotted as a function of tracer concentration, and the data were fitted to the linearized Cheng–Prusoff equation to determine the intracellular inhibition constant (*K*_i_). Washout experiments were performed following a previously described protocol in the presence of 40 nm Kinase Tracer 03 [43].

### 2.4 *In vitro* antiproliferative impact and apoptosis induction

Proliferation experiments were performed in RPMI without phenol red, 10% charcoal‐stripped fetal bovine serum, and in the presence of 0.1 nm R1881, except for LNCaP cells where 1 nm R1881 was added. A dose range of inhibitor concentrations was tested and cell viability measured after 4 days (PC‐3, DU145) or 6 days (VCaP, LNCaP, 22Rv1) using the CellTiter‐Glo luminescent assay (Promega Corporation). For the determination of the combination index (CI), viability assays were performed in starved VCaP cells subsequently treated for 6 days with 0.1 nm R1881 and different ratios of darolutamide and copanlisib or apitolisib. The respective IC_50_ values were calculated and used to determine the CI.

Apoptosis was evaluated following a 48‐h treatment with a dose range of inhibitor concentrations by measuring caspase 3/7 activation (CaspaseGlo 3/7 assay; Promega Corporation) or PARP cleavage (PARP Cleaved 214/215 ELISA assay; Invitrogen/ThermoFisher, Waltham, MA, USA).

The expression of the Bcl‐2‐binding component 3 (BBC3) gene involved in apoptosis was determined using RNA extracted from treated VCaP cells using the TaqMan Fast Advanced Master Mix 4444557 (Applied Biosystems, Waltham, MA, USA) followed by real‐time PCR with primers specific for BBC3 (Hs00248075_m1; Life Technologies, Carlsbad, CA, USA) in a 7900 HT Fast Real‐Time PCR system (Applied Biosystems). Human cyclophilin A was used as endogenous control (4326316E; ThermoFisher).

### 2.5 Sandwich ELISA

H2A.X phosphorylation at serine 139 was determined by sandwich ELISA. Briefly, VCaP cells were grown in RPMI without phenol red, 10% charcoal‐stripped fetal bovine serum for 24 h, and then treated for 48 h with 0.03 nm R1881 and two different concentrations of copanlisib and darolutamide, alone or in combination. Cells were then washed with ice‐cold phosphate‐buffered saline and incubated for 5 min with lysis buffer, scraped, and transferred into sonication tubes. Sonication was performed on ice for 2 min twice, followed by centrifugation. The supernatants were analyzed by sandwich ELISA using the PathScan Phospho‐Histone H2A.X (Ser139) kit #50929, following the manufacturer's instructions (Cell Signaling Technology, Danvers, MA, USA). The chromogenic substrate 3,3′, 5,5′‐tetramethylbenzidine was used to determine peroxidase activity, which was measured at 450 nm absorbance in a Tecan Infinite microplate reader (Tecan Group, Männedorf, Switzerland).

### 2.6 Transcriptomic data analysis

VCaP cells were treated for 24 or 48 h with dimethyl sulfoxide (DMSO) only, 0.1 nm R1881, 0.1 nm R1881 and 1 μm darolutamide, 0.1 nm R1881 and 100 nm copanlisib, or 0.1 nm R1881, 1 μm darolutamide and 100 nm copanlisib. Three samples were profiled for each treatment and time point. RNA isolation and sequencing was performed as previously described [45].

FASTQ reads were mapped via STAR aligner to the human genome GRCh38 and quantified with RSEM (v1.3.0; University of Wisconsin‐Madison, Madison, WI, USA). Only protein‐coding genes with at least five transcripts per million (TPM) in three samples or more were used for further differential gene expression analysis. Differentially expressed genes were identified with DESeq2. Benjamini–Hochberg correction for multiple hypothesis testing was used to define false discovery rate (FDR). Genes with at least an expression fold‐change of 2 and FDR below 10% were considered differentially expressed. Single‐sample gene set enrichment analysis (ssGSEA) was performed with the Gene Set Variation Analysis package from R Bioconductor using TPM values and the hallmark gene sets collection from MSigDB [46].

For comparison with human expression, data from the TCGA Prostate Adenocarcinoma study [47] were downloaded from the Genomics Data Commons portal and differential expression between tumor and normal tissue was assessed with the Wilcoxon test.

### 2.7 *In vivo* efficacy study

Fox Chase SCID mice (CB17/Icr‐*Prkdcscid*/IcrIcoCrl) were obtained from Charles River Laboratories (Wilmington, MA, USA). They were maintained in a Specific Pathogen‐Free vivarium throughout the study. For the efficacy study, 5–8‐week‐old male mice were implanted subcutaneously with LuCaP 35 tumor bits, as previously described [41]. Once tumor volume reached 100 mm^3^, mice were randomized and treatment was started with darolutamide (200 mg·kg^−1^, p.o.; 5‐day on, 2‐day off), copanlisib (14 mg·kg^−1^, i.v.; 2‐day on, 5‐day off) or with both compounds combined. Vehicle and castration groups were used as controls. Subcutaneous tumor growth was measured twice weekly using a digital caliper and tumor volume was calculated using the formula: length × width × height × 0.5236. Animals were euthanized when tumors exceeded 1000 mm^3^, if body weight loss reached 20%, or if animals became otherwise compromised as defined in the University of Washington's Institutional Animal Care and Use Committee protocol 3202‐01. Statistical analysis was performed by ANOVA followed by Dunn's test.

At the end of the *in vivo* phase, tumors were harvested, fixed in formalin and embedded in paraffin. Immunohistochemistry (IHC) analysis was performed on tumor microarrays with 1.5 mm cores which were sectioned (3 μm) and stained with monoclonal antibodies specific for cleaved caspase 3 (clone 5A1E, #9664, 66 ng·mL^−1^; Cell Signaling Technology, Cambridge, UK) or BBC3 (clone E2P7G, #98672, 500 ng·mL^−1^; Cell Signaling Technology).

All animal procedures used in this study were approved by the Institutional Animal Care and Use Committee at the University of Washington in accordance with NIH guidelines. Approved animal license number for the study: University of Washington IACUC protocol 3202‐01.

### 2.8 Data generation and statistical analysis

Biochemical and cell‐based binding studies were performed in at least two independent experiments (*N* ⩾ 2) with two technical replicates each (*n* = 2). Cellular assays were conducted with at least two biological replicates (*N* ⩾ 2) and representative results are shown. Measurement for each treatment condition was done in three different wells (*n* = 3) for the apoptosis and real‐time PCR assays, and in two different wells for the sandwich ELISA (*n* = 2). Statistical analysis was performed using one‐way ANOVA and Dunnett's multiple comparisons test. Differential gene expression of TCGA Prostate Adenocarcinoma data was assessed by the Wilcoxon test.

## 3 Results

### 3.1 PI3K/AKT/mTOR inhibitors with different selectivity profiles inhibit proliferation and induce apoptosis of androgen‐sensitive prostate cancer cell lines

Previous gene expression analysis of VCaP cells treated with the androgen R1881 revealed that beside the expected expression stimulation of androgen target genes, there was also a marked upregulation of the hallmark gene signature of the PI3K/AKT/mTOR pathway [45, 48]. We therefore determined how inhibitors of this pathway with different selectivity profiles affected prostate cancer cell proliferation. PI3K inhibitors, dual PI3K/mTOR inhibitors, and AKT inhibitors were evaluated to have a broad overview. Isoform‐selective effects of the different PI3K inhibitors have previously been described but detailed, comparative studies of binding affinities and kinetics for the essential PI3Kα isoform have so far not been reported. PI3Kα plays a crucial function in many cancer types including prostate cancer, especially in mCRPC where the corresponding gene is frequently amplified [13]. Biochemical kPCA and biophysical SPR assays, and a cellular target engagement assay were applied to establish the PI3Kα binding affinity and kinetic map for selected inhibitors. The steady‐state compound affinities measured with biochemical and biophysical methods were in excellent agreement (Fig. S1A). Also, the comparison between steady‐state *K*_i_ and kinetic *K*_D_ values showed a strong correlation (Fig. S1A). The six compounds investigated bound to PI3Kα with *k*_on_ association rates between 10^5^ and 10^8^ m ^−1^·s^−1^ and *k*_off_ dissociation rates between 10^−3^ and 10^−1^·s^−1^, with residence times between 10 s and 17 min (Fig. S1B). Independently of the method used, copanlisib was one of the fastest equilibrating and slowest dissociating compounds, and it ranked among the most potent PI3Kα binders. Next, we determined the intracellular PI3Kα inhibitor potency in living cells. We used the NanoBRET cellular probe displacement assay and found *K*_i_ values between about 3 and 500 nm for the compounds evaluated, with copanlisib being the most potent one with a *K*_i_ of 3.07 ± 1.18 nm (Fig. S1C). Comparison with the biochemical affinities revealed the cellular affinities to be 2‐ to 10‐fold weaker, most likely due to parameters such as cell permeability, cellular ATP concentrations and intracellular regulatory mechanisms which were not captured in the kPCA or SPR assays. The cellular kinetic displacement assay was then performed in washout mode, and we found that PI3Kα engagement by copanlisib and dactolisib lasted significantly longer than expected from the respective binding rate constants of the compounds (Fig. S1D), suggesting a differentiated mechanism of protracted residence time in living cells. Further investigations of this discrepancy between biochemical and cellular target occupancies led to the discovery of unique micropharmacokinetic properties of copanlisib (not shown) that are currently being further explored.

**Fig. 1:**
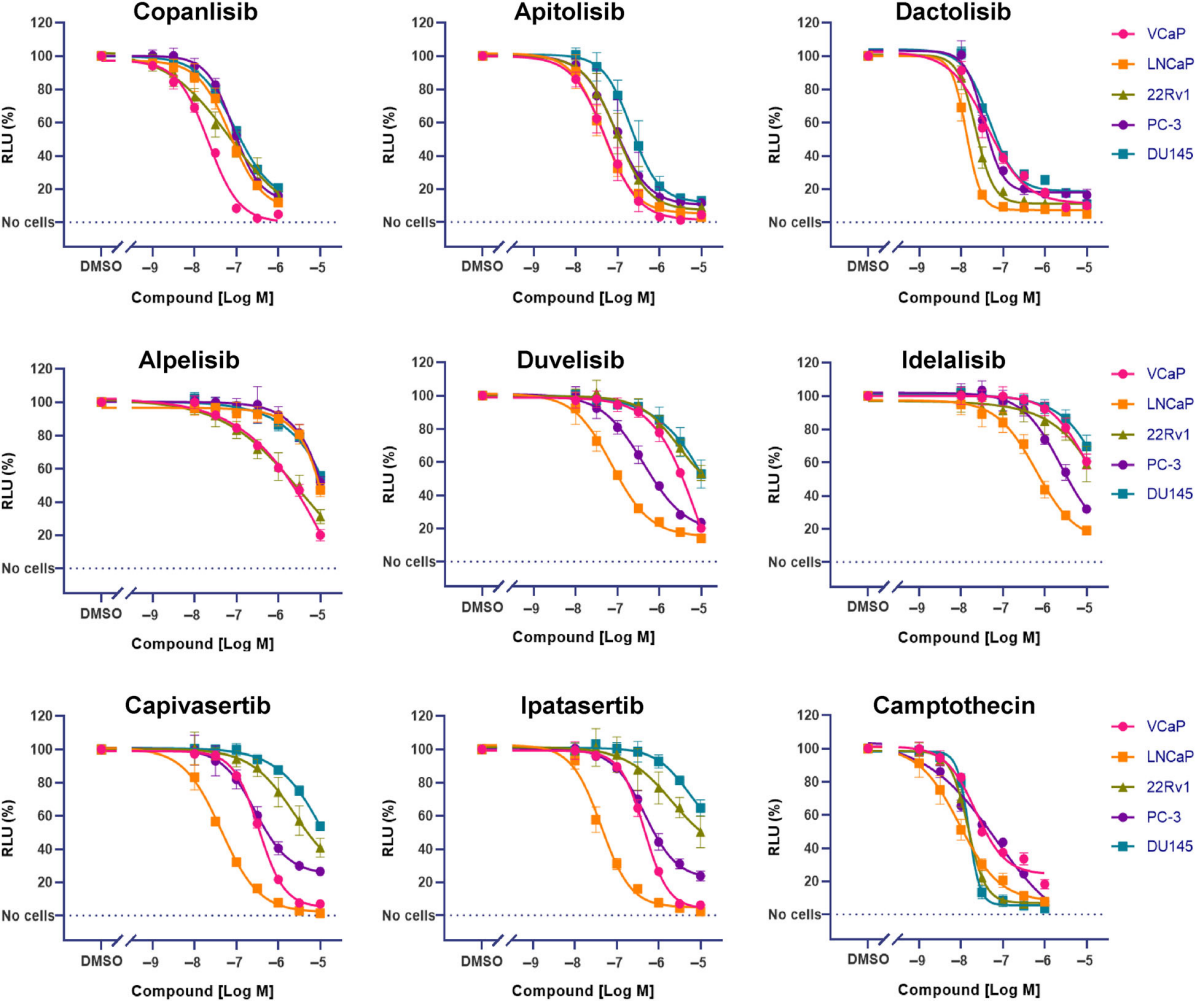
PI3K/AKT/mTOR inhibitors strongly inhibit the proliferation of androgen‐sensitive prostate cancer cells. Viable cell numbers were determined by quantifying ATP. Mean values and standard deviations are shown (*n* = 3). The results are representative of three separate experiments. The cell lines were treated with the indicated compound concentrations for 4 days (PC‐3, DU145) or 6 days (VCaP, LNCaP, 22Rv1). RLU, relative light units.

Copanlisib and dactolisib, and two additional compounds targeting AKT, were then assessed in five prostate cancer cell lines with a different PTEN gene status: wild‐type for VCaP and 22Rv1, heterogenous point mutation for DU145, homozygous deletion for LNCaP and PC‐3 [49, 50, 51]. These cells also exhibit different AKT phosphorylation levels with LNCaP and VCaP cells showing comparatively higher serine 473 phospho‐AKT to AKT signal ratios than 22RV1, PC‐3, or DU145 cells [51, 52, 53]. The pan‐PI3K inhibitor copanlisib and the dual PI3K/mTOR inhibitors apitolisib and dactolisib showed potent antiproliferative effects in all cell lines evaluated (Fig. 1). The AKT inhibitors capivasertib and ipatasertib had a strong impact mainly in androgen‐sensitive cell lines. The dual PI3Kδ/γ inhibitor duvelisib had marked effects in LNCaP cells, whereas the α and δ isoform‐selective compounds alpelisib and idelalisib were generally less active. As a positive control we evaluated the topoisomerase inhibitor camptothecin which inhibited all prostate cancer cell lines tested, as expected (Fig. 1). The IC_50_ values calculated for each compound in each of the five cell lines are shown in Table S1.

**Fig. 2:**
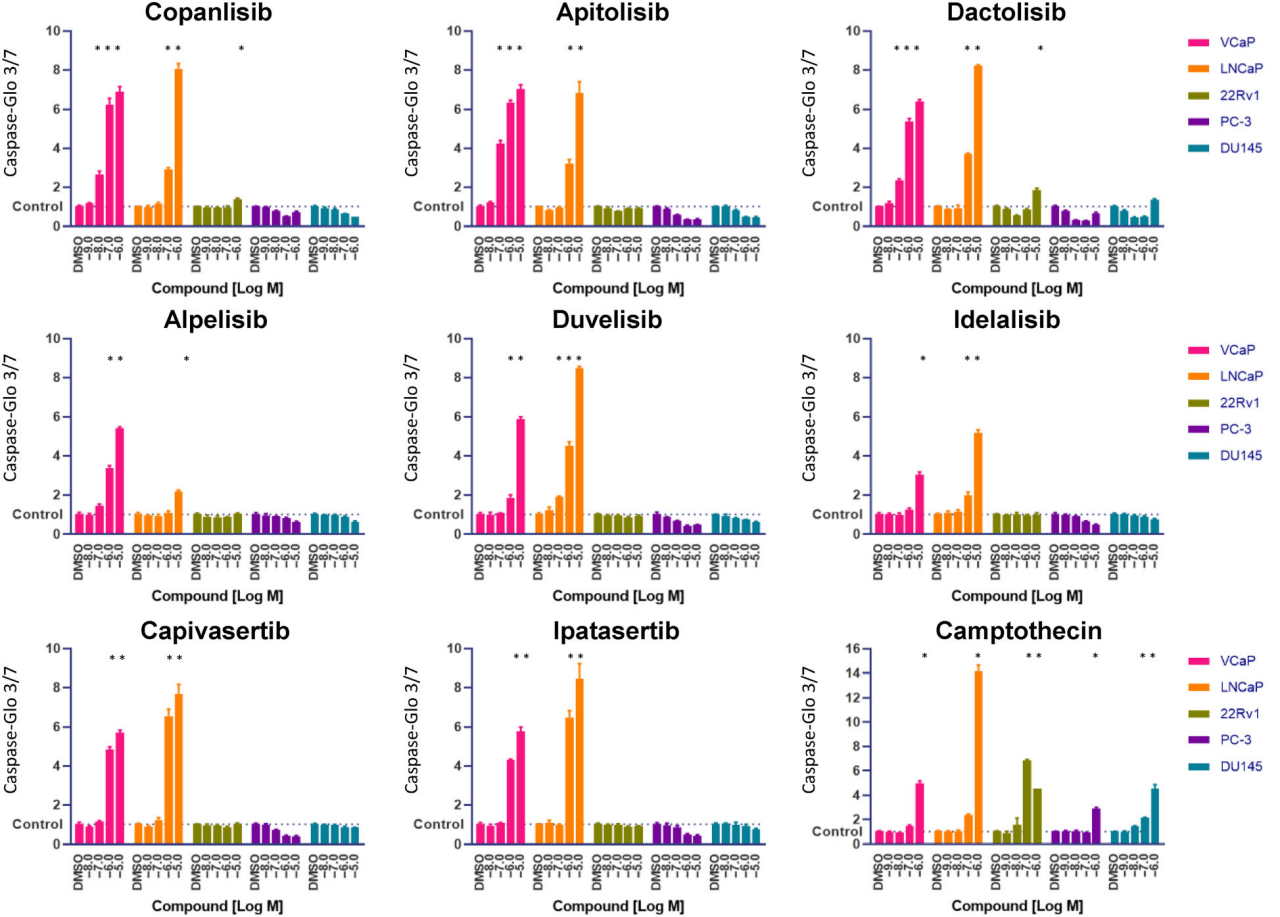
PI3K/AKT/mTOR inhibitors induce strong apoptosis in androgen‐dependent prostate cancer cells. Activation of caspase 3/7 was determined for the cell lines treated with the indicated compound concentrations for 2 days. Mean values and standard deviations are shown (*n* = 3). The results are representative of 3 separate experiments. One‐way ANOVA followed by Dunnett's multiple comparison test was performed to compare treatment with the indicated PI3K/AKT/mTOR inhibitor to the R1881‐only control. Samples with significantly increased apoptotic signals (adjusted *P*‐values below 0.0001) are indicated with *.

We next investigated the effects of the compounds on apoptosis by determining caspase 3/7 activity in the treated cells (Fig. 2). Here also we found that the pan‐PI3K inhibitor copanlisib and the dual PI3K/mTOR inhibitors apitolisib and dactolisib had the strongest effects in the androgen‐sensitive cell lines VCaP and LNCaP. The AKT inhibitors capivasertib and ipatasertib, and the dual PI3Kδ/γ inhibitor duvelisib showed a comparable pro‐apoptotic impact in LNCaP cells and a slightly weaker effect in VCaP cells. The isoform‐selective PI3Kα inhibitors alpelisib and idelalisib were slightly less potent. Apoptosis induction was observed in all cell lines evaluated at the highest camptothecin concentration used (Fig. 2).

### 3.2 Combining PI3K/AKT/mTOR inhibitors with the AR inhibitor darolutamide has pro‐apoptotic effects in androgen‐sensitive prostate cancer cell lines

**Fig. 3:**
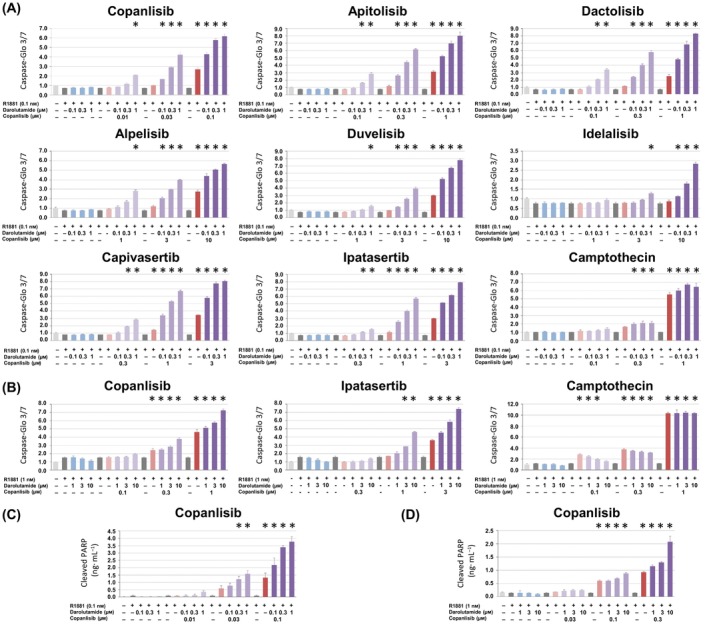
Apoptosis induction in androgen‐dependent prostate cancer cell lines is most pronounced following combined treatment with PI3K/AKT/mTOR inhibitors and darolutamide. (A) VCaP cells and (B) LNCaP cells were treated for 48 h with different compound concentrations and apoptosis was measured by determination of caspase 3/7 activation. (C) VCaP and (D) LNCaP cells were treated with a combination of copanlisib and darolutamide before measuring apoptosis by determination of PARP cleavage. Mean values and standard deviations are shown (*n* = 3). The results are representative of three separate experiments. One‐way ANOVA followed by Dunnett's multiple comparison test was performed to compare treatment with the indicated PI3K/AKT/mTOR inhibitor and/or darolutamide to the R1881‐only control. Samples with significantly increased apoptotic signals (adjusted *P*‐values below 0.0001) are indicated with *.

As the VCaP cell line showed the most pronounced response, we chose it for an in‐depth evaluation of the impact of the combination treatment with PI3K/AKT/mTOR and AR inhibitors. When adding darolutamide to copanlisib or ipatasertib, a dose‐ and time‐dependent increase of caspase 3/7 cleavage was observed, with the 48‐h regimen leading to the most pronounced induction effects (Fig. S2). We then combined darolutamide with different PI3K/AKT/mTOR pathway inhibitors and compared apoptosis induction at 48 h (Fig. 3A). Pronounced effects were observed when combining darolutamide with low copanlisib, apitolisib or dactolisib concentrations (0.01, 0.1 and 0.1 nm, respectively). The co‐treatment with alpelisib, duvelisib or idelalisib also had a significant apoptotic impact, but at higher concentrations (at least 1 nm). This was also the case for the AKT inhibitors capivasertib and ipatasertib where 0.3 nm and higher concentrations were necessary for a significant effect. We performed similar experiments using the LNCaP cell line by combining darolutamide with copanlisib or ipatasertib, as representative PI3K and AKT inhibitors, respectively, and also observed induction of apoptosis (Fig. 3B). These results were confirmed by the evaluation of PARP cleavage in VCaP and LNCaP cells treated with darolutamide and copanlisib (Fig. 3C,D). We then examined a range of genes involved in apoptosis and found that expression of the pro‐apoptotic BBC3 gene was upregulated by copanlisib treatment, and this was most pronounced upon combination with darolutamide in androgen‐stimulated VCaP cells (Fig. S3).

### 3.3 *In vitro* combination treatment with copanlisib and darolutamide has superior antiproliferative activity and comprehensively reverses the androgen response and mTOR transcriptional programs

We then determined the antiproliferative impact of different copanlisib and darolutamide combinations on VCaP cells by measuring viability after 6 days (Fig. S4). Moderate synergistic effects with a CI of 0.82–0.84 were calculated. Comparable results were also obtained when combining the PI3K/mTOR inhibitor apitolisib with darolutamide (Fig. S4).

**Fig. 4:**
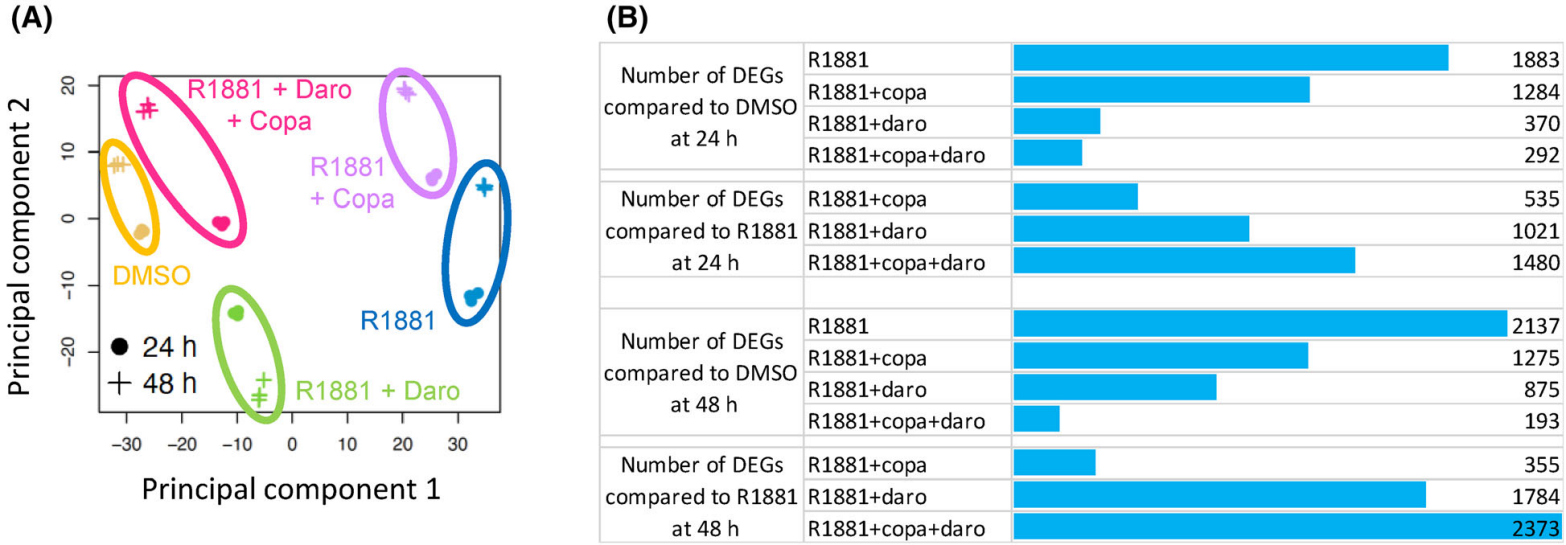
Combination treatment of VCaP cells with copanlisib and darolutamide has a strong transcriptomic impact. The compound concentrations used were 0.1 nm R1881, 100 nm copanlisib, 1 μm darolutamide. (A) Principal component analysis based on 1000 most variable genes. (B) Number of differentially expressed genes (DEGs) upon indicated treatments compared to the DMSO or R1881 groups at 24 and 48 h. Copa, copanlisib; Daro, darolutamide.

Next, we performed a transcriptomic study of the impact of copanlisib and darolutamide as single agents or in combination, at two different time points, on the strongly androgen‐sensitive VCaP cell line. The results of principal component analysis (PCA) are outlined in Fig. 4A. A clear effect of androgen stimulation was visible along the first principal component, with a strong difference between DMSO‐treated and R1881‐stimulated samples. The combined R1881 and copanlisib treatment was only marginally different from R1881, while the R1881 and darolutamide as well as the R1881, darolutamide and copanlisib samples were closer to the DMSO control along the first principal component. The combined R1881, darolutamide and copanlisib treatment was the closest to the DMSO control, indicating the most efficient suppression of R1881‐induced transcriptional changes, which is consistent with the superior effect on proliferation observed. The difference between the time points was aligned along the second principal component for all the treatments. Pairwise comparison of gene expression changes between the different conditions revealed that the combined treatment with R1881, darolutamide and copanlisib led to the lowest number of genes being differentially expressed compared with DMSO (Fig. 4B). This number decreased over time, suggesting strong time‐dependent inhibition of androgen‐induced transcription (Fig. 4B).

**Fig. 5:**
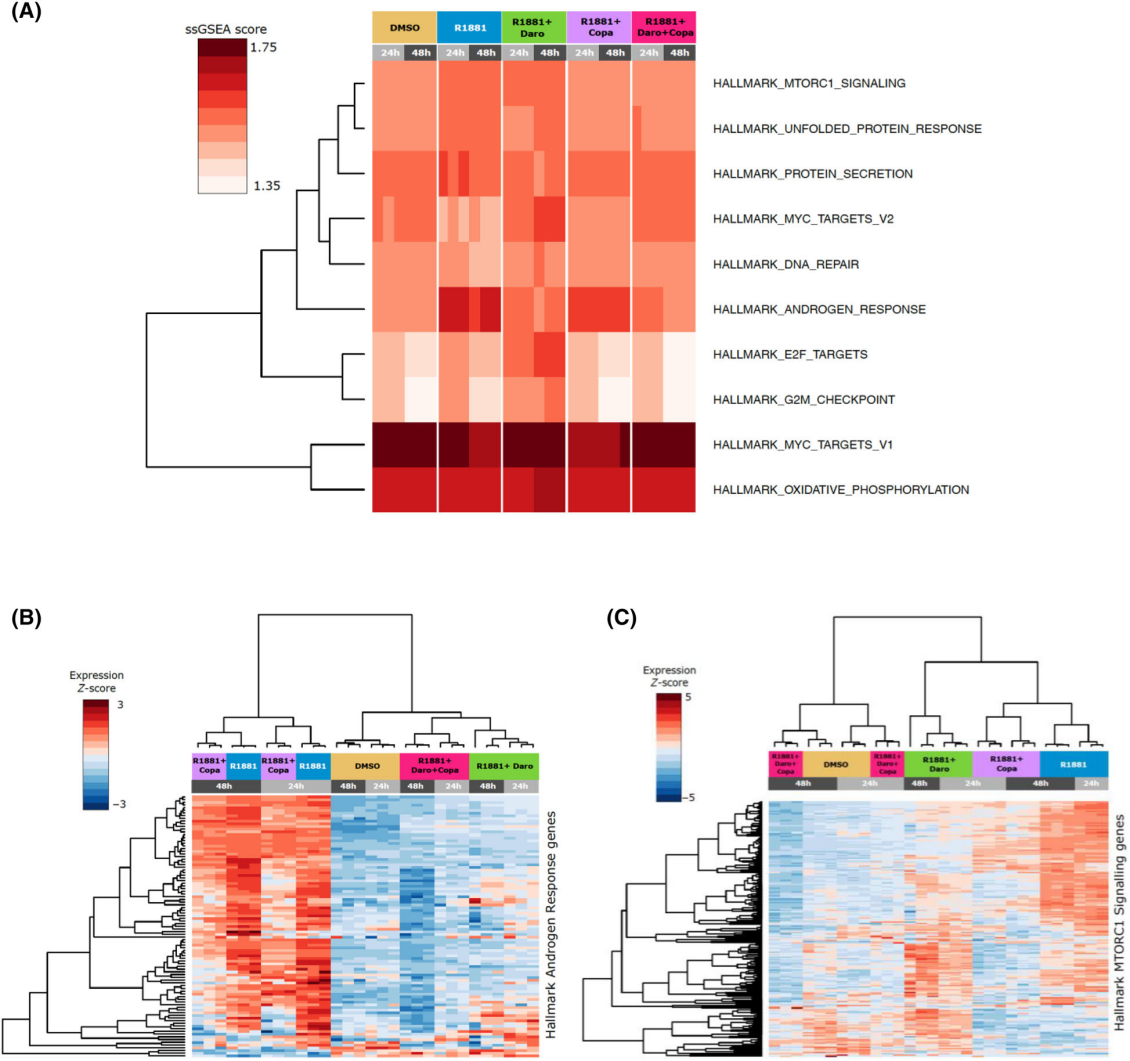
*In vitro* combination treatment with copanlisib and darolutamide comprehensively reverses the androgen response and mTOR transcriptional programs. (A) Heatmap showing single‐sample gene set enrichment analysis (ssGSEA) scores for the most impacted hallmark genesets. Gene sets with enrichment above 1.5 in at least one sample are shown. (B) Heatmap and hierarchical clustering showing expression of genes from the hallmark androgen response geneset. (C) Heatmap and hierarchical clustering showing expression of genes from the hallmark mTORC1 signaling geneset.

In order to understand the pathways affected, we performed ssGSEA on all the samples using the hallmark gene sets collection from MSigDB. We selected gene sets with enrichment above 1.5‐fold in at least one sample for further visualization and exploration. The enrichment of selected hallmark gene sets was visualized in a heatmap (Fig. 5A). The results show that the androgen response hallmark was clearly upregulated by R1881 treatment as expected. The addition of copanlisib alone had only a moderate effect on androgen response gene set enrichment, while darolutamide and the darolutamide plus copanlisib cotreatment notably decreased it. Hierarchical clustering of the expression of genes from the hallmark androgen response gene set revealed a clear clustering of the copanlisib‐treated condition with the androgen‐stimulated one, while the darolutamide and the darolutamide plus copanlisib combination conditions assembled near the DMSO control, which demonstrates the efficient inhibition of classical androgen target genes by either of these treatments (Fig. 5B). We also observed a notable regulation of the hallmark gene set of mTORC1 signaling. It was upregulated by androgen; however, the addition of darolutamide alone did not suppress it, while copanlisib alone or in combination with darolutamide efficiently downregulated it (Fig. 5A). This was also evident upon hierarchical clustering of the expression of genes from this set (Fig. 5C). This analysis revealed that despite most of the mTORC1 pathway genes being regulated by androgen, only the combination of copanlisib and darolutamide reverted this feature close to the profile seen in the DMSO control, whereas the individual darolutamide or copanlisib treatment had less impact, highlighting the additional effect of the combination.

The described effects were exemplified by comparing the expression levels of individual genes in the treated groups. Androgen response gene set representatives encoding acyl CoA synthetase long‐chain family member 3 (ACSL3), FK506‐binding protein prolyl isomerase 5 (FKBP5), transmembrane protease, serine 2 (TMPRSS2) and mTORC1 pathway representatives encoding elongation of very long chain fatty acid protein 6 (ELOVL6), emopamil‐binding protein (EBP), and 7‐dehydrocholesterol reductase (DHCR7) are shown (Fig. S5A,B).

Notably, gene expression of mTOR itself and of its direct effectors, the translation initiation factors 4 [54], was upregulated by androgen and strongly repressed by darolutamide and copanlisib combination treatment (Fig. S6A). We also examined the expression of mTOR and of the eukaryotic translation initiation factors (EIF) 4E2 and 4G1 in clinical samples and found it to upregulated in prostate cancer compared with normal prostate tissue (Fig. S6B), which further supports the role of this pathway in prostate cancer oncogenesis. Concerning AR expression, similar transcript levels were observed in the androgen plus darolutamide, and also in the androgen plus darolutamide and copanlisib group, slightly lower than those seen in the DMSO only group (Fig. S5C).

### 3.4 *In vitro* combination treatment with copanlisib and darolutamide stimulates H2A.X serine 139 phosphorylation

**Fig. 6:**
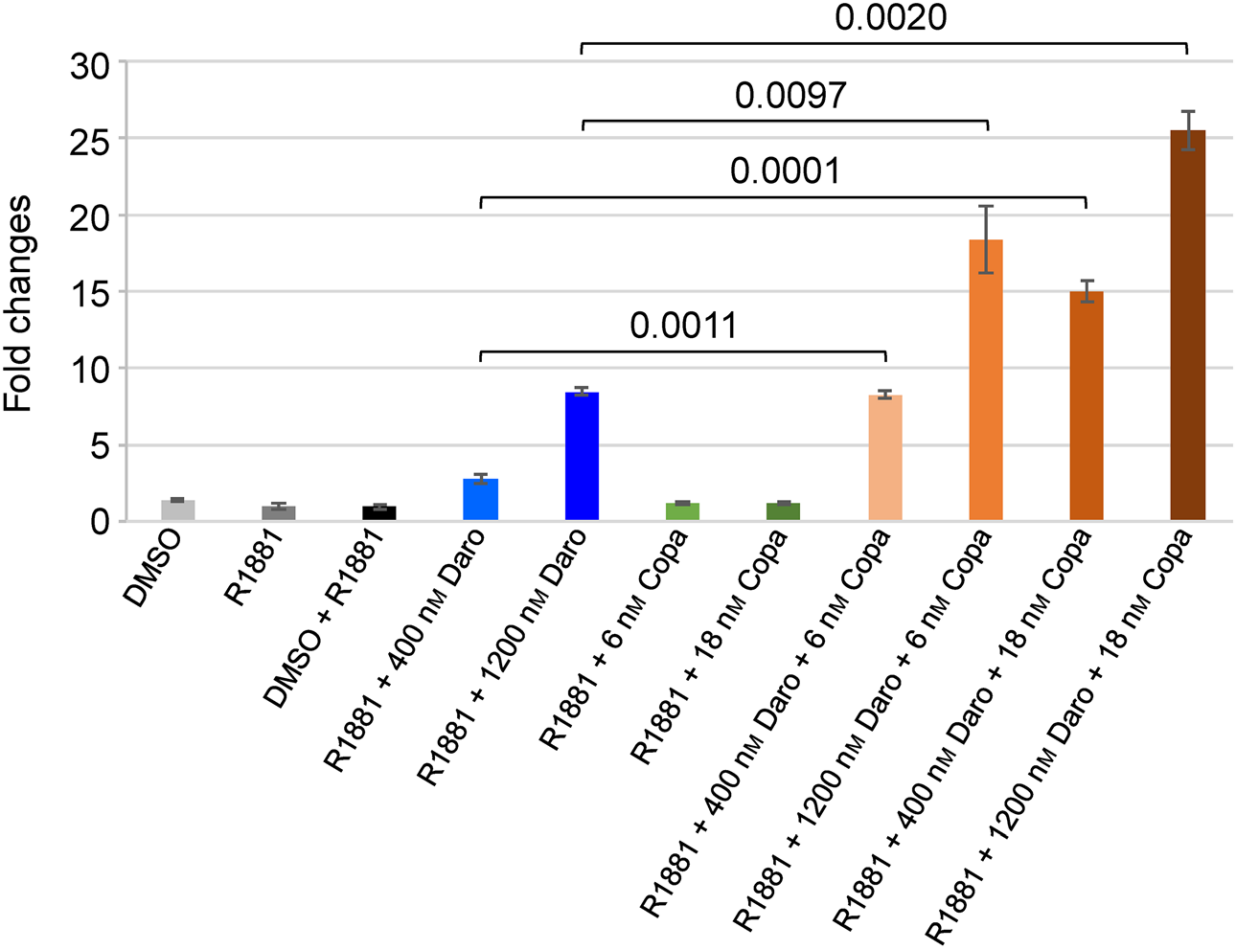
Combination treatment with copanlisib and darolutamide stimulates H2A.X serine 139 phosphorylation. Sandwich ELISA results of VCaP cells treated for 48 h as indicated. Staining was performed with an antiphospho‐Ser139‐histone H2A.X antibody. Mean values and standard deviations are shown (*n* = 2). The results are representative of two separate experiments. One‐way ANOVA followed by Dunnett's multiple comparison test was performed to compare combination treatments with the respective darolutamide‐only treatment. Significant differences with the corresponding adjusted *P*‐values are indicated. Copa, copanlisib; Daro, darolutamide.

AR function is directly linked to faithful DNA repair via regulation of DNA damage response genes [55, 56, 57]. As we observed a marked impact of the copanlisib and darolutamide combination on cell viability, we additionally determined the impact of the treatments on DNA double‐strand breaks by measuring H2A.X serine 139 phosphorylation (Fig. 6). The results show that individual copanlisib application had no impact on H2A.X phosphorylation. Conversely, darolutamide treatment increased H2A.X phosphorylation, especially at the higher concentration used. The effect was significantly enhanced when darolutamide was combined with copanlisib, indicating that DNA double‐strand breaks are involved in the antiproliferative effects we observed.

### 3.5 *In vivo* combination treatment with darolutamide and copanlisib enhances antitumor efficacy and leads to apoptosis

**Fig. 7:**
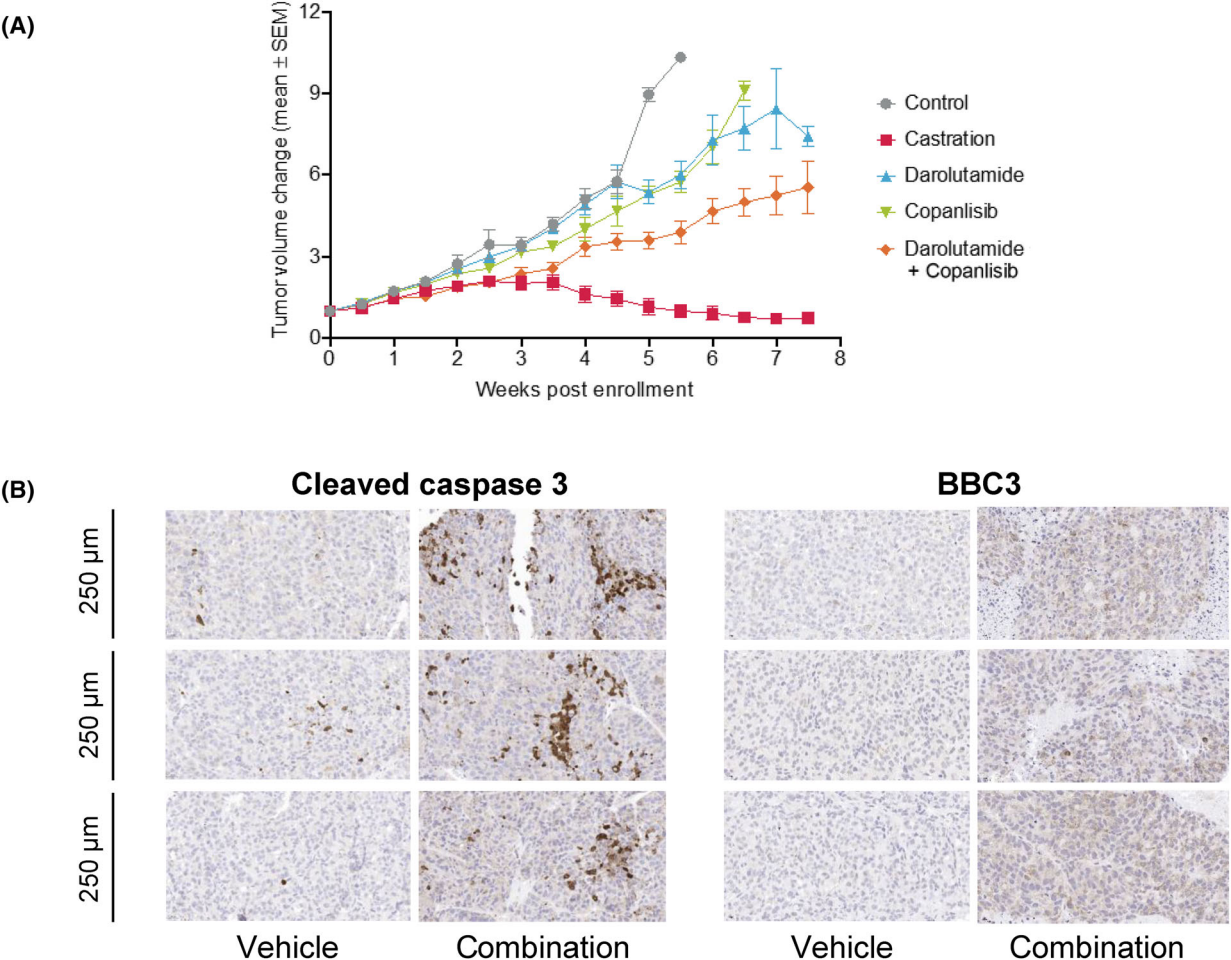
Combination treatment with darolutamide and copanlisib enhances antitumor efficacy and leads to apoptosis *in vivo*. (A) SCID male mice bearing the LuCaP 35 PDX tumors were treated with darolutamide (200 mg·kg^−1^, p.o.; 5‐day on, 2‐day off), copanlisib (14 mg·kg^−1^, i.v.; 2‐day on, 5‐day off) or their combination. Vehicle and castration control groups were used for comparison. The following numbers of mice were included: 18 (control), 14 (castration), 15 (darolutamide), 15 (copanlisib), 23 (darolutamide + copanlisib). Tumor volume was measured twice weekly. The combination treatment showed improved efficacy when compared to darolutamide or copanlisib monotherapies (*P* = 0.0017 and 0.0075, respectively, at 5 weeks). Statistical analysis was performed by ANOVA followed by Dunn's test. (B) Tissue microarrays were prepared with 1.5 mm cores from tumors collected at the end of the *in vivo* study (*n* = 5 per group), sectioned (3 μm), and stained for the indicated targets. Representative tissue sections stained for cleaved caspase 3 or Bcl‐2‐binding component 3 (BBC3) are shown. The same enlargement was used for all images. Scale bars are indicated.

We then set out to investigate whether the synergistic effects observed translated to the *in vivo* situation. First, small studies were conducted with multiple prostate cancer PDX models in order to identify those most likely to respond to the combination treatment (data not shown). We selected the LuCaP 35 PDX model due to its moderate response to individual copanlisib or darolutamide treatment. It expresses wild‐type AR and harbors a biallelic PTEN deletion [58]. Single‐compound regimen had only a minor impact on tumor growth (Fig. 7A). The darolutamide and copanlisib cotreatment showed significantly superior efficacy to monotherapy from 5 weeks after dosing start (Fig. 7A). Immunohistochemistry analysis of tumor samples taken at the end of the experiment revealed increased levels of cleaved caspase 3 and BBC3 in the combination group, compared with the vehicle group (Fig. 7B).

## 4 Discussion

In the present work, we have shown that the pan‐PI3K inhibitor copanlisib ranked among the strongest binders and inhibitors of the main PI3K isoform α and that it displayed superior antiproliferative efficacy in multiple prostate cancer cell lines, regardless of their PTEN status or AKT phosphorylation state. Apoptosis induction was observed mainly in VCaP and LNCaP cells for most inhibitors tested. This is possibly linked to the elevated activation state of the PI3K/AKT/mTOR pathway in these two cell lines, as evidenced by their high serine 473 phospho‐AKT levels [51, 52, 53]. Copanlisib already induced apoptosis in these two cell lines at 10 nm, and also at lower concentrations when combining it with darolutamide. Moreover, the DNA damage response was also increased following darolutamide treatment and more so after cotreatment with copanlisib, in line with previous data reporting that antiandrogens downregulate the expression of DNA repair genes [55, 56, 57]. The detailed transcriptomic analysis we performed additionally revealed that genes from the androgen response hallmark were strongly regulated by the cotreatment. Prominent examples include genes coding for ACSL3 and DBI/ACBP, which are both involved in steroidogenesis [59, 60], and FKBP5, an essential cofactor involved in AR dimerization [61]. Also, mTORC1 hallmark genes involved in lipid and cholesterol biosynthesis [62, 63, 64] were markedly downregulated, further supporting the role of these metabolic pathways in prostate cancer progression [65, 66]. Most importantly, our results showed that the combined treatment with copanlisib and darolutamide also had a significant inhibitory effect on the growth of the PDX prostate cancer model LuCaP 35 and induced apoptosis *in vivo*.

Previous studies with inhibitors of PI3K or mTOR combined with AR signaling inhibitors have reported their impact on individual prostate cancer cell lines [23, 53, 67, 68]. An early work claimed that the PI3Kβ isoform was the essential driver in PTEN‐deficient prostate cancer [69], but this was not confirmed in a larger cohort of patient‐derived models, suggesting shifts in isoform dependency [26]. Concerning AKT inhibitors, *in vitro* treatment with capivasertib reduces cell viability of several prostate cancer cell lines [24, 70]. *In vivo* studies document that combining capivasertib with the first‐generation AR inhibitor bicalutamide delays LNCaP tumor progression [24], whereas the combination with the more potent AR inhibitor enzalutamide induces regression [70]. Another report shows that cotreatment with dactolisib and enzalutamide leads to regression of LNCaP xenografts and strongly reduces tumor sizes in Pten^lox/lox^ mice with established prostate tumors [23]. A study combining the AR antagonist apalutamide with the pan‐AKT inhibitor GSK690693 shows superior activity in mouse‐derived prostate cancer cell lines and also *in vivo* in a castration‐naïve Pten‐deficient mouse model [71]. Our present study extends the previous data by showing the superior efficacy of a pan‐PI3K inhibitor combined with an AR inhibitor and by providing a detailed analysis of the molecular impact on androgen‐sensitive prostate cancer cell lines.

As outlined above, clinical studies revealed that on‐target toxicities represent an important challenge for the effective therapy of CRPC with PI3K/AKT/mTOR pathway inhibitors, so selecting compounds with the right profile will be essential. Intermittent drug dosing has led to improved tolerability but at the cost of incomplete inhibition of the pathway [72]. Our systematic characterization of the mechanistic and cellular effects of PI3K/AKT/mTOR pathway inhibitory compounds with different profiles and the evaluation of the synergistic effects with the AR inhibitor darolutamide offer a rational starting approach for the selection of the combination pair best suited for the treatment of prostate cancer patients. Clearly, achieving maximal efficacy while maintaining an acceptable side effect profile remains a challenge that needs to be approached from different angles and will necessitate the identification of stratification and pharmacodynamic biomarkers.

## 5 Conclusions

Reciprocal crosstalk between the PI3K/AKT/mTOR and AR pathways represents an important mechanism involved in prostate cancer progression. The present work shows that combining the pan‐PI3K inhibitor copanlisib with the AR inhibitor darolutamide leads to potent proliferation inhibition of androgen‐sensitive prostate cancer cell lines while inducing DNA double‐strand breaks and apoptosis. Our detailed transcriptomic analysis additionally reveals a strong impact of the dual treatment on androgen response and mTORC1 hallmark gene sets. The superior efficacy of the combination treatment is further confirmed in a prostate cancer PDX model that responds only marginally to single agents. Altogether these results will provide important guidance for future studies aiming at evaluating the impact of combination treatments with AR signaling inhibitors and PI3K/AKT/mTOR inhibitors in prostate cancer patients.

## Conflict of interest

TS, EN, SH, RL, OvA, SG, SJB, VG, VP, AF‐M, OP, DM, and BH are or were employees of Bayer AG. EN, RL, SG, DM, and BH are stockholders of Bayer AG. EN, RL, OvA, SG, SJB, OP, DM, and BH are inventors on Bayer AG patent applications. EC obtained funding under Institutional Sponsored Research Agreements from Genentech, Sanofi, AbbVie, Astra Zeneca, Foghorn Pharmaceuticals, Kronos Bio, MacroGenics, Janssen Research, Bayer Pharmaceuticals, Forma Pharmaceuticals, Gilead, Zenith Epigenetics and is consultant of DotQuant. JDV and MBR are employees of Promega, which owns patents related to target engagement.

## Author contributions

EN, OvA, SG, EC, AF‐M, DM, and BH were involved in conceptualization. TS, SH, AB, RL, HMN, SJB, VG, VP, JDV, and MBR were involved in experiments and investigation. TS, EN, SH, RL, EC, AF‐M, JDV, MBR, OP, DM, and BH were involved in formal analysis. TS, SH, AB, RL, HMN, SJB, and VG were involved in methodology. TS, EN, SH, HMN, AF‐M, and BH were involved in visualization. EN and BH were involved in writing—original draft. EN, SH, OvA, SG, HMN, EC, VP, AF‐M, OP, DM, and BH were involved in writing—review & editing.

### Peer review

The peer review history for this article is available at https://www.webofscience.com/api/gateway/wos/peer‐review/10.1002/1878‐0261.13577.

## Supporting information


**Table S1.** Calculated IC_50_ for the antiproliferative activity of different PI3K/AKT/mTOR inhibitors in prostate cancer cell lines.
**Fig. S1.** Target binding affinity and kinetic parameters of selected PI3Kα inhibitors.
**Fig. S2.** Time‐dependent induction of apoptosis.
**Fig. S3.** Copanlisib combined with darolutamide increases the expression of the pro‐apoptotic Bcl‐2‐binding component 3 (BBC3) gene.
**Fig. S4.** Combined treatment of VCaP cells leads to additive antiproliferative effects.
**Fig. S5.** Impact of copanlisib and darolutamide treatment on the expression levels of selected genes in VCaP cells.
**Fig. S6.** Expression of mTOR and its immediate effectors in VCaP cells treated with copanlisib, darolutamide or their combination, and in normal and cancerous prostate tissue data from the TCGA project.

## Data Availability

The authors confirm that all data underlying the findings are fully available without restriction. The transcriptomic data are available under GSE245454.
