# Supplementary material for: Dual targeting of the androgen receptor and PI3K/AKT/mTOR pathways in prostate cancer models improves antitumor efficacy and promotes cell apoptosis
Source: Mol Oncol. 2024 Jan 15;18(3):726–42. doi: 10.1002/1878-0261.13577 (PMC10920092; doi:10.1002/1878-0261.13577)
Supplement: Supplementary file 1 — Table S1. Calculated IC50 for the antiproliferative activity of different PI3K/AKT/mTOR inhibitors in prostate cancer cell lines. Fig. S1. Target binding affinity and kinetic parameters of selected PI3Kα inhibitors. Fig. S2. Time‐dependent induction of apoptosis. Fig. S3. Copanlisib combined with darolutamide increases the expression of the pro‐apoptotic Bcl‐2‐binding component 3 (BBC3) gene. Fig. S4. Combined treatment of VCaP cells leads to additive antiproliferative effects. Fig. S5. Impact of copanlisib and darolutamide treatment on the expression levels of selected genes in VCaP cells. Fig. S6. Expression of mTOR and its immediate effectors in VCaP cells treated with copanlisib, darolutamide or their combination, and in normal and cancerous prostate tissue data from the TCGA project. [file MOL2-18-726-s001.pdf]

Compound		Cell line / IC_{50} (nM)				
**Name**	**Selectivity**	**VCaP**	**LNCaP**	**22Rv1**	**PC-3**	**DU145**
Copanlisib (BAY 80-6946)	Pan-PI3K	7	34	53	81	88
Apitolisib (GDC-0980)	Pan-PI3K/mTOR	19	24	91	102	223
Dactolisib (BEZ235)	Pan-PI3K/mTOR	15	10	20	34	52
Alpelisib (BYL719)	PI3Kα	216	5,168	1,968	8,425	9,457
Duvelisib (IPI-145)	PI3Kδ/γ	1,082	34	7,605	470	7,453
Idelalisib (GS-1101)	PI3Kδ	3,683	324	9,985	2,685	10,000
Capivasertib (AZD5363)	Pan-AKT	155	17	3,613	402	8,623
Ipatasertib (GDC-0068)	Pan-AKT	221	18	6,915	565	10,000
Camptothecin	Topoisomerase I	14	3	13	27	14

Table S1. Calculated IC_{50} for the anti-proliferative activity of different PI3K/AKT/mTOR inhibitors in prostate cancer cell lines.

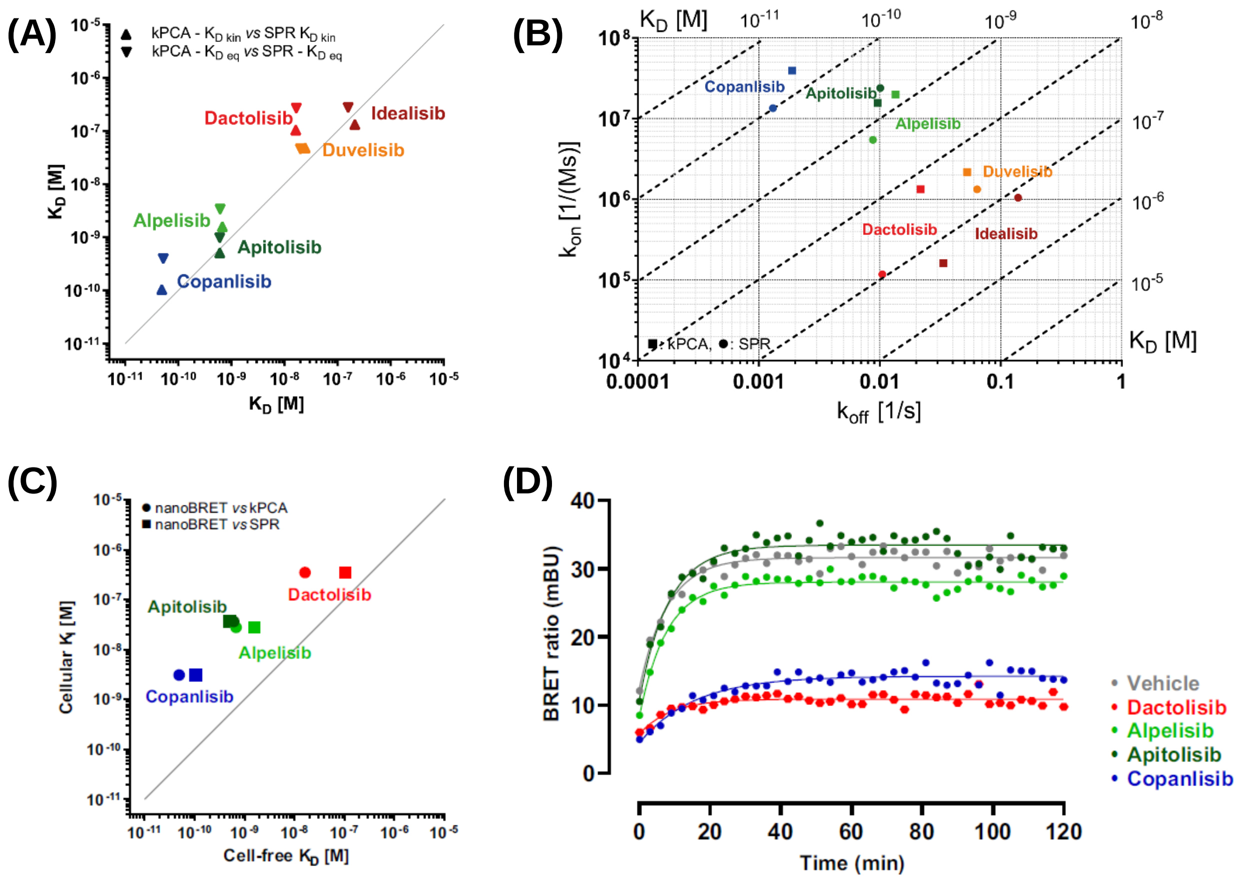

Fig. S1. Target binding affinity and kinetic parameters of selected PI3Kα inhibitors. (A) Correlation between the steady-state compound affinities (K_D) determined by the kPCA (x-axis) and SPR (y-axis) technologies. (B) Rate plot with isoaffinity diagonals (RaPID) of the inhibitors characterized by kPCA or SPR, with off- and on-rates for individual compounds represented respectively on the graph‘s x- and y-axes, and affinities readable from the dotted diagonal lines. (C) Comparison of cell-free affinity (K_D) values determined (x-axis) and intracellular affinity (K_I) values (y-axis) determined using a probe displacement assay based on the NanoBRET technology. (D) Real-time monitoring of intracellular probe displacement after drug washout for estimation of the apparent residence time of inhibitors in living cells. The dots represent the NanoBRET signals corresponding to the interaction between the fluorescent probe and the NanoLuc-PI3Kα fusion protein (y-axis) that are recovered over time upon compound removal (x-axis). The solid lines represent the fit of the data to an exponential decay model from which half-lives of the inhibitor-protein complexes were derived. Biochemical and cell-based binding studies were performed in at least two independent experiments (N⩾2) with two technical replicates each (n=2). Cellular assays were conducted with at least two biological replicates (N⩾2) and representative results are shown.

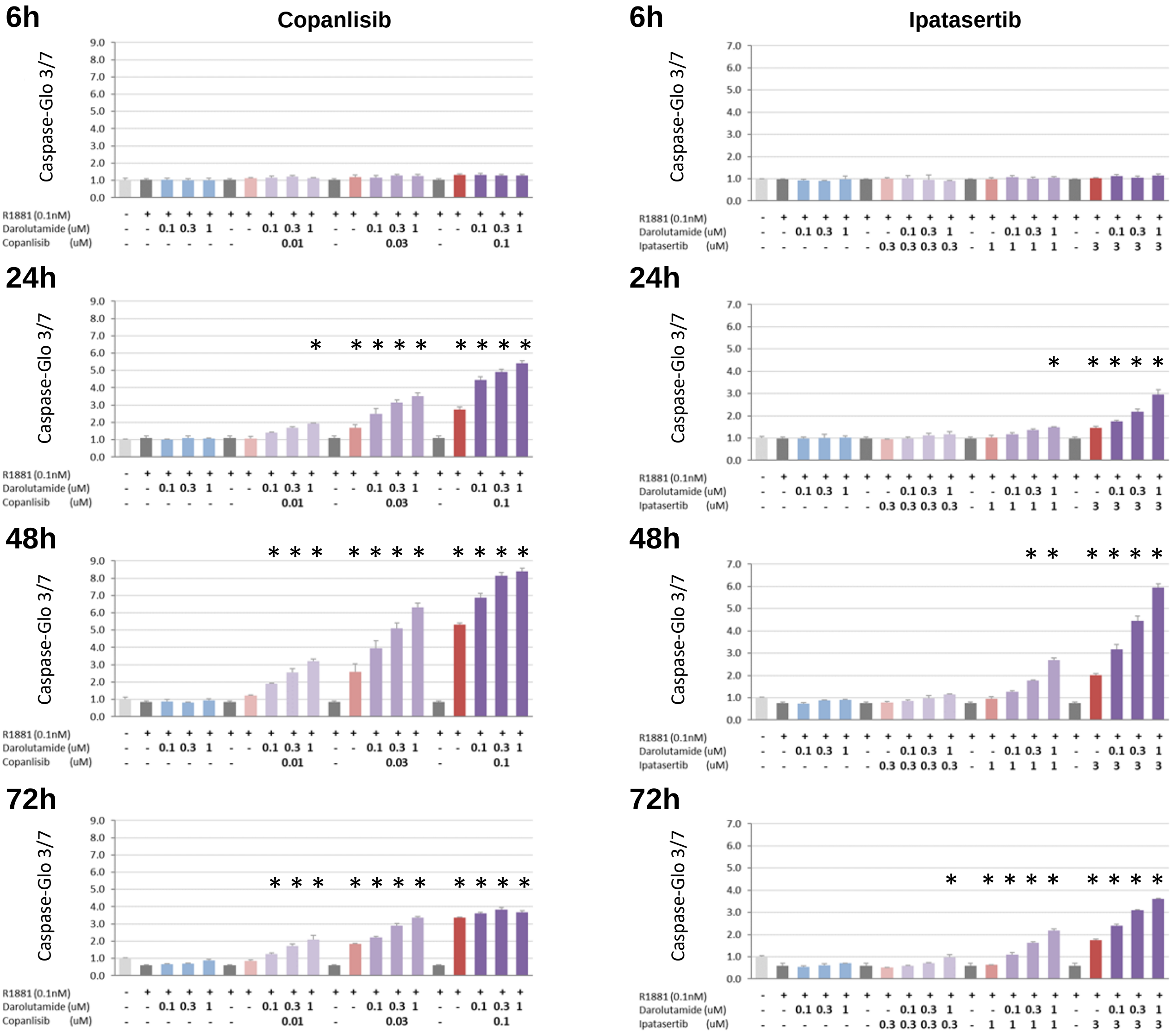

Fig. S2. Time- and dose-dependent induction of apoptosis is observed in VCaP cells co-treated with copanlisib or ipatasertib plus darolutamide. Apoptosis was determined by measuring caspase 3/7 activity. Mean values and standard deviations are shown (n=3). The results are representative of 3 separate experiments. One-way ANOVA followed by Dunnett´s multiple comparison test was performed to compare treatment with the indicated PI3K/AKT/mTOR inhibitor and/or darolutamide to the R1881-only control. Samples with significantly increased apoptotic signals (adjusted P-values below 0.0001) are indicated with *.

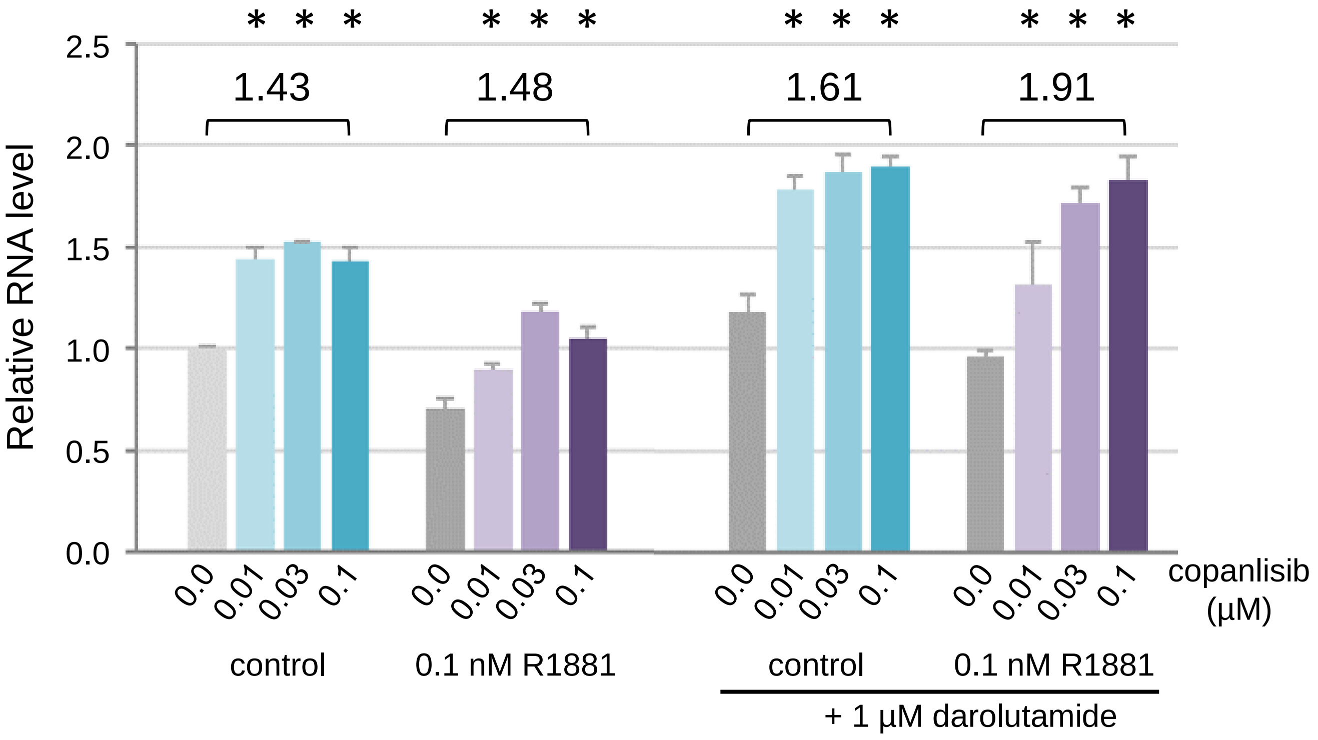

Fig. S3. Copanlisib combined with darolutamide increases the expression of the pro-apoptotic Bcl-2-binding component 3 (BBC3) gene. VCaP cells were treated with the indicated compound concentrations for 24 hours before RNA extraction and determination of BBC3 expression by real-time PCR. Average of $2^{-\Delta\Delta Ct}$ values and corresponding standard deviations are shown (n=3). The results are representative of 2 separate experiments. One-way ANOVA followed by Dunnett´s multiple comparison test was performed to compare the treated samples with their respective control. Samples with significantly increased expression (adjusted P-values below 0.0001) are highlighted with *. Fold inductions for the highest copanlisib-treated groups and their respective controls are indicated.

Copanlisib **Apitolisib**

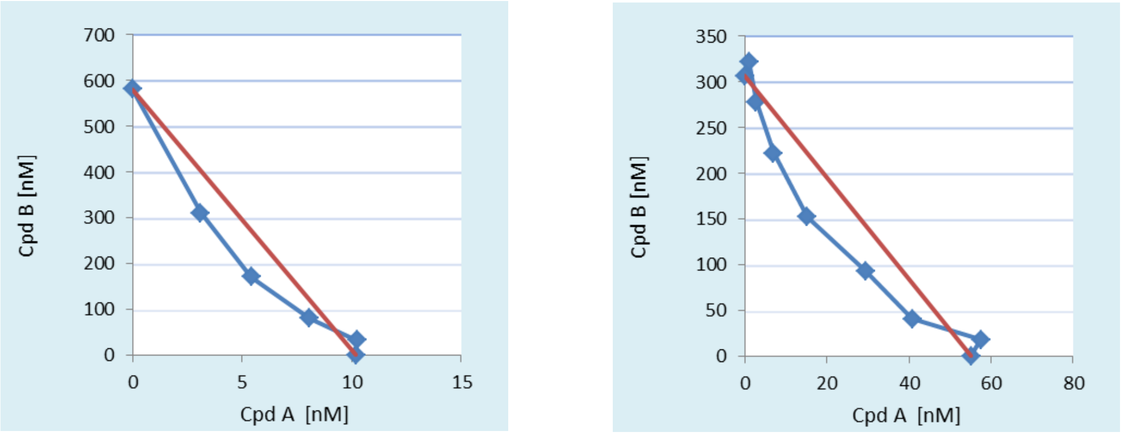

Fig. S4. Combined treatment of VCaP cells leads to additive anti-proliferative effects. Cells were treated with different concentrations of copanlisib or apitolisib (Cpd A) and darolutamide (Cpd B) for 6 days in the presence of 0.1 nM R1881 and cell viability measured. Combination index (CI) values of 0.82-0.84 and 0.77-0.84 were calculated.

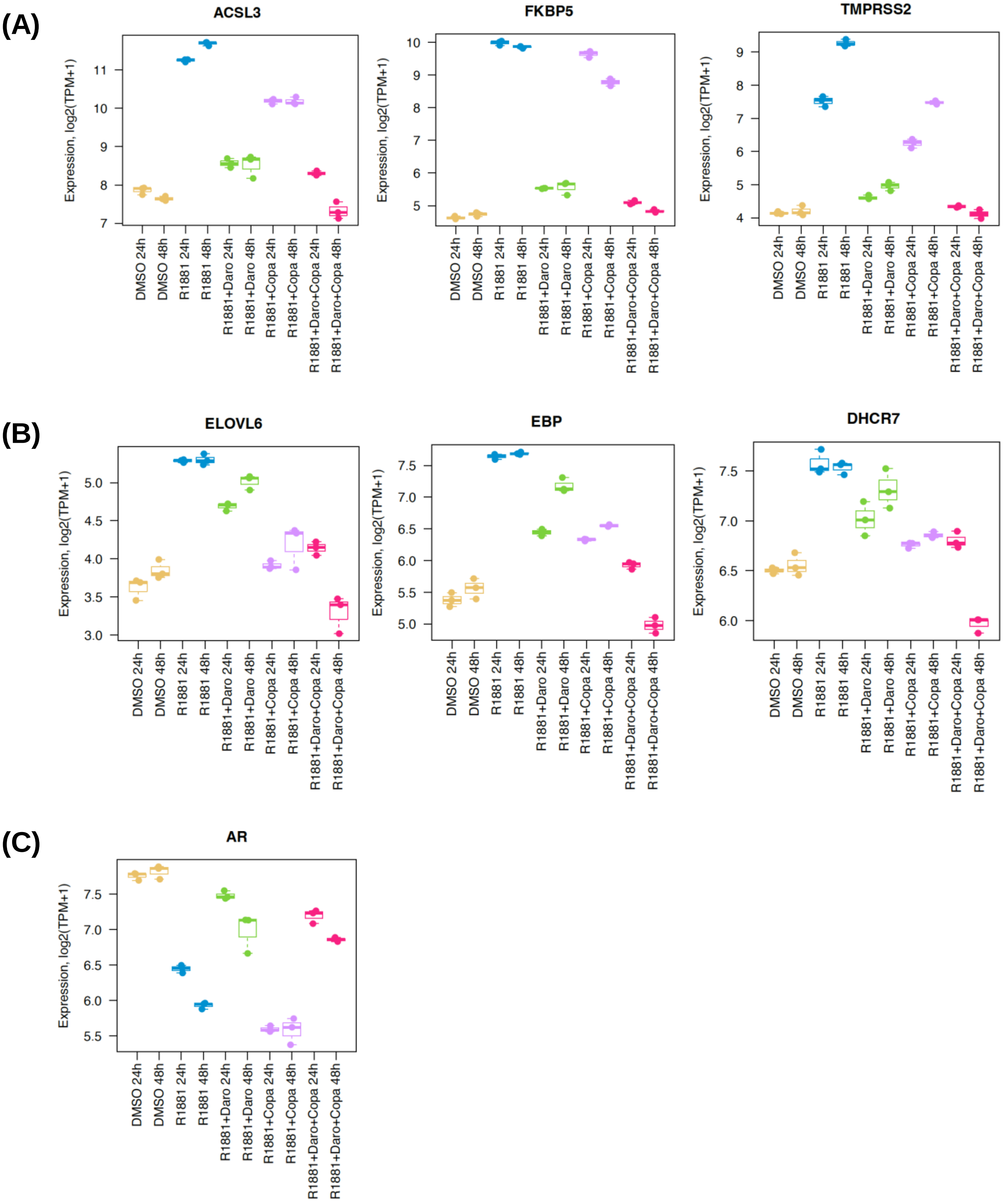

Fig. S5. Changes in gene expression levels in VCaP cells following treatment with copanlisib, darolutamide or their combination. (A) Examples of regulated genes from the androgen receptor pathway: ACSL3, FKBP5, TMPRSS2. (B) Examples of regulated genes from the mTORC1 pathway: ELOVL6, EBP, DHCR7. (C) Expression levels of AR. Daro: darolutamide; Copa: copanlisib.

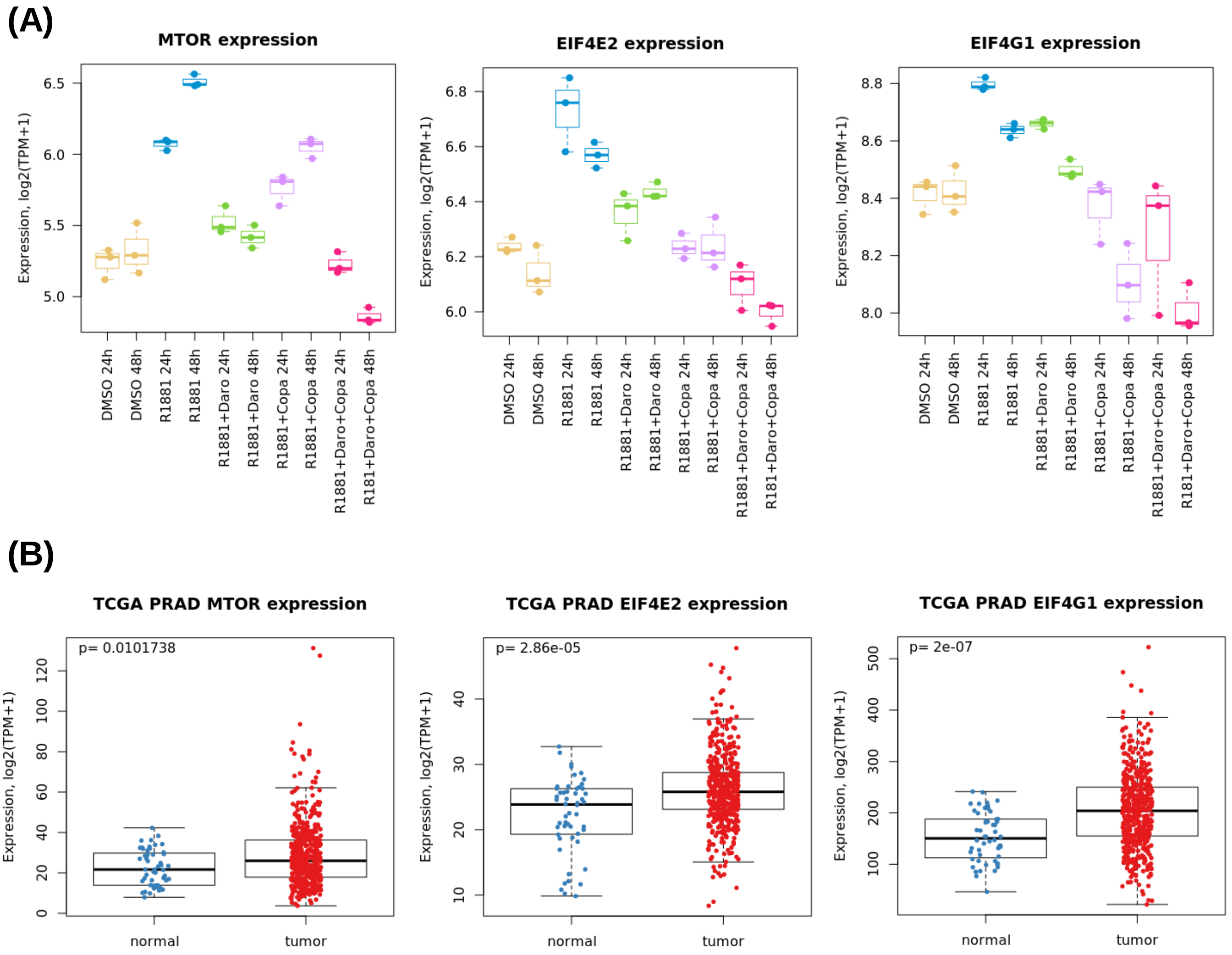

Fig. S6. Expression of mTOR and its immediate effectors, the eukaryotic translation initiation factors EIF4E2 and EIF4G1. (A) Expression in VCaP cells following treatment with darolutamide (Daro), copanlisib (Copa), or their combination for 24 or 48 hours. (B) Expression in normal and cancerous prostate tissue. Human data originate from the TCGA Prostate Adenocarcinoma study. Differential expression was assessed with the Wilcoxon test.
